# Familial Mediterranean Fever With Compound Heterozygosity for the Pyrin Variant L110P-E148Q: A Case Report

**DOI:** 10.7759/cureus.96542

**Published:** 2025-11-11

**Authors:** Shuto Fukuda, Tetsuhito Muranaka, Yutaro Otsuka, Yunosuke Takishin, Yasuyuki Kunieda

**Affiliations:** 1 Department of Internal Medicine, Wakkanai City Hospital, Wakkanai, JPN

**Keywords:** compound heterozygosity, familial mediterranean fever, fever of unknown origin, genetic disease, mefv gene

## Abstract

Familial Mediterranean fever (FMF) is a monogenic autoinflammatory disorder caused by *MEFV* mutations. Exon 10 variants such as M694V are high-penetrance mutations associated with severe phenotypes, colchicine resistance, and amyloidosis. In contrast, exon 2 variants, particularly E148Q, are modifying alleles that require compound heterozygosity to manifest disease symptoms. Japanese patients frequently present with exon 2-related genotypes, resulting in milder phenotypes and a lower prevalence compared with Mediterranean populations.

This report describes the case of a 30-year-old Japanese male with a three-month history of recurrent febrile episodes lasting 5-10 days, accompanied by fatigue and anorexia but no serositis. Elevated C-reactive protein and serum amyloid A levels were observed. The family history included periodic fevers in his mother and maternal grandmother. Genetic analysis revealed compound heterozygosity for *MEFV* exon 2 variants L110P-E148Q. Colchicine therapy induced complete remission, similar to his mother who also carried the same mutations.

FMF is markedly less common in Japan than in Mediterranean regions, reflecting different genetic architectures. FMF can develop with a single exon 10 mutation in Mediterranean patient cohorts, whereas exon 2-exon 10 or exon 2-exon 2 compound heterozygosity is needed in Japanese patients. Owing to the absence of serositis in this patient, the disease could have been misdiagnosed without genetic testing. Thus, recognizing population-specific patterns is essential for appropriate management. This case demonstrates that exon 2 variants, although traditionally considered benign, can cause FMF in compound heterozygous conditions. Accordingly, genetic testing beyond exon 10 is critical for Japanese patients with recurrent fever.

## Introduction

Familial Mediterranean fever (FMF) is one of the most common monogenic autoinflammatory diseases, caused by pathogenic variants in the *MEFV* gene, which encodes pyrin. FMF is most prevalent in Mediterranean populations (approximately 1 in 200 to 1 in 1,000 individuals) and markedly less common in East Asia, including Japan, where its exact prevalence remains undefined. We have also specified that no comprehensive nationwide epidemiological data are currently available for Japan. FMF represents the prototypical autoinflammatory disease, characterized by recurrent episodes of fever and polyserositis [1]. The *MEFV* gene, located on chromosome 16p13.3, consists of 10 exons with pathogenic variants predominantly clustered in exons 2 and 10 [1].

Exon 10 variants represent the most clinically notable FMF variants. These variants occur within the B30.2/SPRY domain, which is responsible for caspase-1 activation [2]. The most common exon 10 variants include M694V, M680I, and M694I, which are all hypermorphic variants that decrease the activation threshold of the pyrin inflammasome [3], resulting in uncontrolled IL-1β production and enhanced inflammatory responses. This pathogenic effect demonstrates a gene-dosage relationship, wherein more exon 10 pathogenic variants result in a stronger pyrin inflammasome response [3]. The most prevalent variant in Mediterranean populations, M694V, exhibits high penetrance and is associated with severe phenotypic manifestations such as high fever, splenomegaly, and musculoskeletal conditions [1,4].

On the other hand, exon 2 variants, particularly E148Q, have a more complex pathogenic profile. Although E148Q was historically considered a benign polymorphism, recent evidence has recognized it as a potentiating variant that enhances inflammasome activation *in vitro* [5]. E148Q exhibits an additive effect when present in *cis* form with known pathogenic FMF variants, suggesting a modifying rather than primary pathogenic role [5]. Japanese population studies revealed that E148Q alone cannot sufficiently cause clinical manifestations, with affected individuals exhibiting no episodes of fever or serositis [6]. However, when combined with exon 10 variants such as M694I, the compound heterozygous state exhibits a typical FMF phenotype with remarkably elevated IL-18 levels during afebrile phases [6].

Patients harboring exon 10 variants typically exhibit more severe clinical phenotypes. High-penetrance variants, particularly M694V, are associated with earlier disease onset, more frequent attacks, and higher complication rates (e.g., amyloidosis) [1,4]. Symptoms typically include high-grade fever (>95%), peritonitis (60%-70%), pleuritis (35%-40%), and arthritis (30%-35%) [7]. Additionally, exon 10 variants also demonstrate less favorable responses to colchicine therapy and induce a higher risk of developing amyloid A amyloidosis, which is the most serious long-term complication of FMF [1]. Accordingly, this increased inflammatory burden necessitates the use of more aggressive therapeutic interventions, including IL-1 inhibitors in colchicine-resistant cases. In contrast, isolated exon 2 variants typically exhibit milder phenotypes or cause incomplete disease penetrance. The E148Q variant requires compound heterozygosity with pathogenic exon 10 variants to manifest the typical symptoms of FMF [6]. This pattern is particularly evident in Japanese populations, wherein 19.8% of patients with FMF have the E148Q/M694I combination, but those with isolated E148Q or M694I variants are asymptomatic [6,7]. Therefore, it is difficult to diagnose cases of FMF with variants in exons other than exon 10, requiring caution in clinical practice. This report describes a case of FMF with compound heterozygosity for the pyrin variant L110P-E148Q that was successfully diagnosed and treated.

This article was previously presented as a meeting abstract at the 304th Hokkaido Regional Meeting of the Japanese Society of Internal Medicine on June 28, 2025.

## Case presentation

A 30-year-old Japanese male had been experiencing recurrent febrile episodes of approximately 38°C lasting about 5-10 days over three consecutive months, which were being managed with symptomatic treatment at another hospital. The fever pattern was noted, with the febrile episodes recurring every 3-4 weeks, each lasting 5-10 days, accompanied by fatigue and anorexia. One month later, he presented to our department for further evaluation after experiencing another four-day episode of fever. During each episode of fever, the patient reported decreased appetite and fatigue, but no abdominal or chest pain. His medical history includes bronchial asthma and sinusitis, and he was being treated for atopic dermatitis. He smokes 20 cigarettes per day and consumes an average of 30 g of alcohol per day. At the initial visit, the patient had stable vital parameters (temperature: 37.4°C, blood pressure: 121/65 mmHg, pulse rate: 98 bpm, and SpO2: 98%) with no remarkable skin findings. Initial laboratory tests revealed elevations in lactate dehydrogenase (258 IU/L), C-reactive protein (2.46 mg/dL), and serum amyloid A protein (83.7 mg/L) (Table 1). Both antinuclear antibody and antineutrophil cytoplasmic antibody tests were negative. Chest X-ray, abdominal ultrasound, and echocardiography revealed no evidence of pleural, pericardial, or peritoneal effusions.

**Table 1:** Laboratory data Serum amyloid A levels and C-reactive protein levels were abnormally elevated compared to the reference range.

Test name	Observed value	Reference range
White blood cell count	3,200/μL	6,500-8,200/μL
Neutrophils	53.2%	35%-75%
Lymphocytes	30.4%	16.5%-49.5%
Monocytes	14.2%	2%-10%
Eosinophils	1.9%	0%-5%
Basophils	0.3%	0%-2.5%
Red blood cell count	474×10^4^/μL	430-560×10^4^/μL
Hemoglobin level	13.7 g/dL	13.5-17.0 g/dL
Platelet count	26.5×10^4^/μL	15.0-35.0×10^4^/μL
Prothrombin time-international normalized ratio	1.12	0.9-1.1
Activated partial thromboplastin time	37.8 sec	24.0-39.0 sec
Total protein level	6.9 g/dL	6.5-8.2 g/dL
Albumin level	3.7 g/dL	3.7-5.5 g/dL
Aspartate aminotransferase	28 IU/L	8-38 IU/L
Alanine aminotransferase	30 IU/L	4-44 IU/L
Lactate dehydrogenase	258 IU/L	124-222 IU/L
Gamma-glutamyl transpeptidase	29 IU/L	16-84 IU/L
Alkaline phosphatase	56 IU/L	38-113 IU/L
Total bilirubin	0.3 mg/dL	0.2-0.8 mg/dL
Blood urea nitrogen	9 mg/dL	8-20 mg/dL
Serum creatinine level	0.75 mg/dL	0.65-1.30 mg/dL
Serum sodium level	141 mEq/L	136-148 mEq/L
Serum potassium level	4.2 mEq/L	3.6-5.0 mEq/L
Serum chloride level	105 mEq/L	95-108 mEq/L
Serum calcium level	8.9 mg/dL	8.5-10.5 mg/dL
Serum ferritin level	381 ng/mL	21-282 ng/mL
C-reactive protein level	2.46 mg/dL	0-0.4 mg/dL
Fasting plasma glucose	96 mg/dL	65-105 mg/dL
Glycated hemoglobin	6.2%	4.6%-6.2%
Serum amyloid A level	83.7 mg/L	0-3.0 mg/L
Protein in urine	(-)	(-)
Hematuria	(-)	(-)
Urinary glucose	(-)	(-)

**Figure 1:**
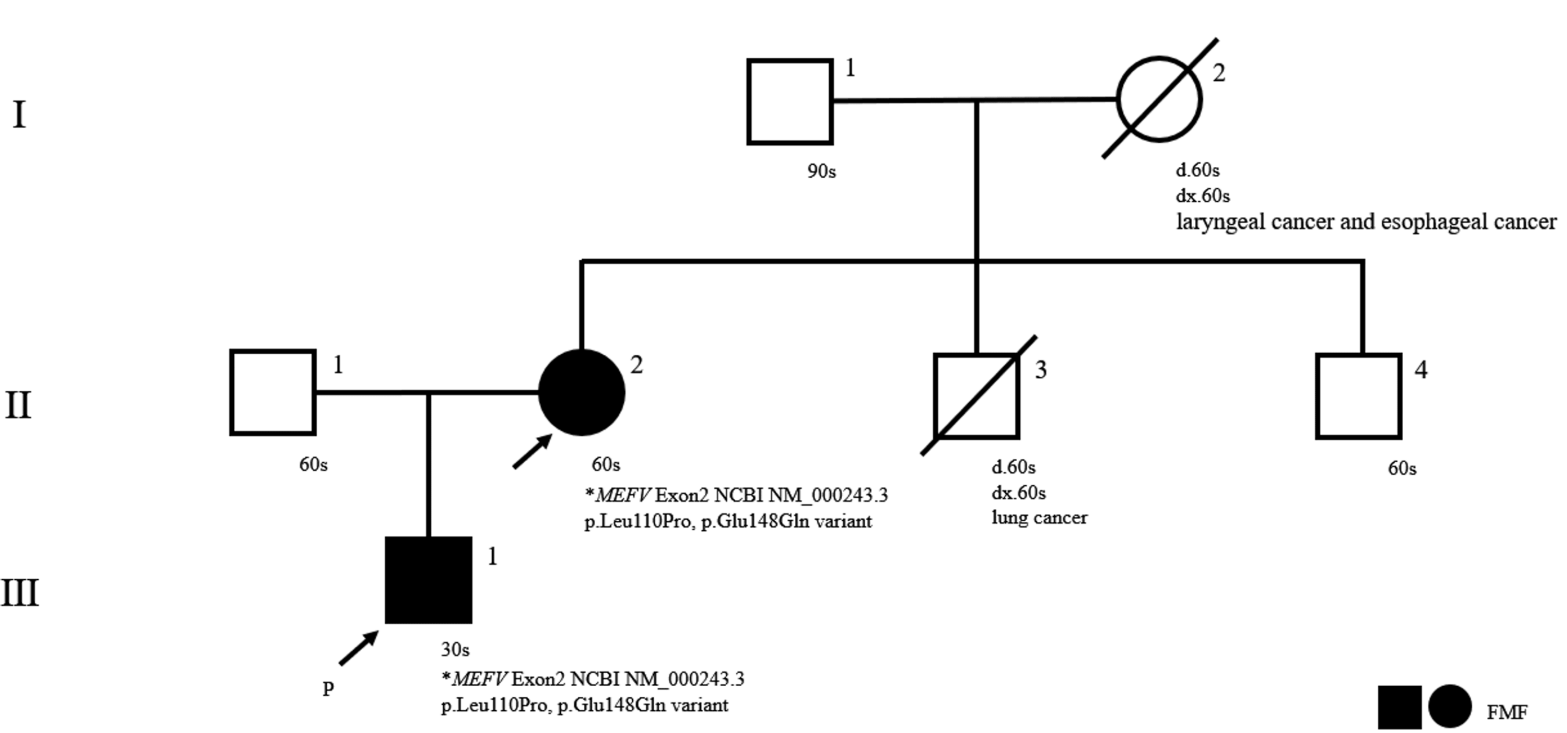
Pedigree This chart shows the family history of this patient. “p” indicates the proband who led to the discovery of the affected family, and the arrows indicate the genetic counseling clients. Black circles or squares indicate individuals genetically diagnosed with familial Mediterranean fever. The maternal grandmother had similar clinical symptoms but passed away due to another disease. *Based on the reference sequence from the National Center for Biotechnology Information (NCBI)

Family history revealed similar monthly febrile episodes in his mother and maternal grandmother, who had passed away from laryngeal and esophageal cancer (Figure 1). 

FMF with mutations in exons other than exon 10 was suspected. Treatment challenge was initiated with regular colchicine administration, and genetic counseling was provided. *MEFV* gene analysis revealed L110P-E148Q, a compound heterozygous mutation in *MEFV* exon 2 (NCBI NM_000243.3), confirming the diagnosis of FMF caused by exon 2 mutations. The positive genetic history was identified by genetic testing performed after genetic counseling, revealing the same *MEFV* variants in the patient’s mother. Both the patient and his mother were placed on regular oral colchicine at a dose of 0.5 mg twice daily (1.0 mg/day total) and have not experienced any periodic fever episodes since then. Therefore, they were managed with colchicine monotherapy, and biologic agents were not required.

## Discussion

The prevalence of FMF is highest among Mediterranean populations, with Turkey showing an overall prevalence of 1:1000, reaching up to 1:500 in certain regions [1]. Israel reports a prevalence of 1-2:1000, with distinct mutation patterns. In particular, M694V is predominant in non-Ashkenazi Jews (76.8%), whereas E148Q is more common in Ashkenazi populations [1]. Armenian populations also have similar prevalence rates of 1-2:1000, with carrier frequencies of 1:5 [1]. The mutation spectrum in Mediterranean countries reflects ancient founder effects, wherein high-penetrance exon 10 mutations (M694V and M680I) are sufficient for disease manifestation. Environmental factors in these regions may also contribute to more severe disease expression and higher rates of amyloidosis compared to emigrant populations [1]. In contrast, FMF is remarkably less common in Japan, with an estimated 292 total patients (95% CI: 187-398), resulting in a prevalence of approximately 2.3 per million population [7]. This prevalence is 400-fold lower than that of Mediterranean countries. This could be attributed to markedly different genetic architecture, with compound heterozygosity (M694I + E148Q) being essential for disease manifestation [6,7]. Japanese patients exhibit distinct clinical characteristics, including a later age of disease onset (37.3% after age 20), lower amyloidosis rates (3.7%), and excellent response to low-dose colchicine (mean: 0.89 mg/day) [7]. Another report also found that 36% were aged 20 or older, with only one case of amyloidosis among 80 patients [8]. Furthermore, there have been reports of late-onset cases even with Exon 10 mutations [9]. Additionally, Migita K reported that more than half of the Japanese FMF patients without MEFV exon 10 mutations presented with an atypical FMF phenotype, indicating that Japanese FMF patients tend to be divided into two phenotypes by a variation of MEFV mutations [10]. This requirement for compound heterozygosity suggests differences in evolutionary pressures and genetic backgrounds compared to Mediterranean populations. Therefore, if clinicians focus solely on the clinical symptoms of FMF in exon 10, there is a risk of missing the diagnosis of FMF caused by other exon mutations.

In this case, although there were periodic fevers, there were no symptoms of serositis typical of FMF with exon 10 mutation. The presence of periodic fever alone warrants consideration for other genetic disorders such as tumor necrosis factor receptor-associated periodic syndrome, hyper-IgD syndrome, or cryopyrin-associated periodic fever syndrome, as well as connective tissue diseases such as juvenile idiopathic arthritis or adult-onset Still's disease [11-14]. Our patient was initially managed with colchicine, avoiding any invasive treatment. The absence of joint pain or rash made the diagnosis of other connective tissue diseases less likely. Afterward, FMF was promptly diagnosed by testing the *MEFV* gene. However, in the absence of *MEFV* gene mutations, investigation of mutations in other genes (*TNFRSF1A*,* MVK* genes, and so on) is necessary.

FMF is one of the most common monogenic autoinflammatory disorders, representing the prototypical syndrome of monogenic autoinflammatory recurrent fever. Despite being a well-known autoinflammatory disease, evaluations of the pathogenesis and genetic background of FMF reveal a deeper complexity than previously recognized. Nevertheless, the accurate interpretation of genetic test results is critical for the timely diagnosis and appropriate treatment of patients with FMF [15]. We emphasize that timely genetic evaluation for these patients not only enables accurate diagnosis but also serves as a guide for family counseling, risk assessment, and treatment planning.

## Conclusions

Exon 10 mutations function as primary pathogenic variants with high penetrance and severe phenotypes, whereas exon 2 mutations serve as modifying variants that require compound heterozygosity for disease manifestation. Geographic variations in FMF can be attributed to distinct genetic architectures, with Mediterranean populations showing high-penetrance single mutations and Japanese populations requiring compound heterozygosity. These considerations are important in genetic counseling, diagnostic approaches, and therapeutic strategies for different populations. Understanding these mutation-specific and population-specific patterns is essential to optimize the clinical management and develop personalized therapeutic approaches for patients with FMF worldwide.

## References

[1] Lancieri M, Bustaffa M, Palmeri S (2023). An update on familial Mediterranean fever. Int J Mol Sci.

[2] Tufan A, Lachmann HJ (2020). Familial Mediterranean fever, from pathogenesis to treatment: a contemporary review. Turk J Med Sci.

[3] Miyashita K, Matsuda Y, Okajima M, Toma T, Yachie A, Wada T (2022). Role of E148Q in familial Mediterranean fever with an exon 10 mutation in MEFV. Pediatr Int.

[4] Bilge ŞY, Solmaz D, Şenel S (2019). Exon 2: is it the good police in familial Mediterranean fever?. Eur J Rheumatol.

[5] Migita K, Uehara R, Nakamura Y (2012). Familial Mediterranean fever in Japan. Medicine (Baltimore).

[6] Migita K, Izumi Y, Jiuchi Y (2016). Familial Mediterranean fever is no longer a rare disease in Japan. Arthritis Res Ther.

[7] Kishida D, Yazaki M, Nakamura A, Tsuchiya-Suzuki A, Shimojima Y, Sekijima Y (2020). Late-onset familial Mediterranean fever in Japan. Mod Rheumatol.

[8] Tsuchiya-Suzuki A, Yazaki M, Nakamura A, Yamazaki K, Agematsu K, Matsuda M, Ikeda S (2009). Clinical and genetic features of familial Mediterranean fever in Japan. J Rheumatol.

[9] Fujita Y, Ogawa S, Sumichika Y (2025). Elderly-onset familial Mediterranean fever carrying MEFV exon 10 variants in a Japanese patient. Intern Med.

[10] Migita K, Agematsu K, Yazaki M (2014). Familial Mediterranean fever: genotype-phenotype correlations in Japanese patients. Medicine (Baltimore).

[11] Assrawi E, Louvrier C, El Khouri E (2022). Mosaic variants in TNFRSF1A: an emerging cause of tumour necrosis factor receptor-associated periodic syndrome. Rheumatology (Oxford).

[12] Schutt C, Siegel DM (2023). Autoinflammatory diseases/periodic fevers. Pediatr Rev.

[13] Sönmez HE, Bayındır Y, Batu ED (2023). Cardiovascular manifestations of monogenic periodic fever syndromes. Clin Rheumatol.

[14] Vomero M, Lamberti L, Corberi E (2025). Specialized pro-resolving mediators and autoimmunity: recent insights and future perspectives. Autoimmun Rev.

[15] Batu ED, Basaran O, Bilginer Y, Ozen S (2022). Familial Mediterranean fever: how to interpret genetic results? How to treat? A quarter of a century after the association with the Mefv gene. Curr Rheumatol Rep.

